# Anti-Obesity Properties of *Boesenbergia rotunda* Rhizome Extract: Regulation of Inflammation, Lipid Metabolism, and Insulin Signaling in *ob/ob* Mice

**DOI:** 10.3390/molecules30030501

**Published:** 2025-01-23

**Authors:** Muhammad Hermawan Widyananda, Dinia Rizqi Dwijayanti, Airi Fujii, Keita Minamisaka, Yuto Nishidono, Mikio Nishizawa, Nashi Widodo

**Affiliations:** 1 Department of Biology, Faculty of Mathematics and Natural Sciences, Universitas Brawijaya, Malang 65113, East Java, Indonesia; mh.hermawan@student.ub.ac.id (M.H.W.); rd.dinia@ub.ac.id (D.R.D.); nishizaw@sk.ritsumei.ac.jp (M.N.); 2 Research Center of Complementary Medicine and Functional Food, Universitas Brawijaya, Malang 65113, East Java, Indonesia; 3 Asia-Japan Research Institute, Ritsumeikan Asia-Japan Research Organization, Ritsumeikan University, Ibaraki 567-8570, Osaka, Japan; 4 College of Life Sciences, Ritsumeikan University, Kusatsu 525-8577, Shiga, Japan; fujii-ai@fc.ritsumei.ac.jp (A.F.);; 5 Research Organization of Science and Technology, Ritsumeikan University, Kusatsu 525-8577, Shiga, Japan; nisidono@fc.ritsumei.ac.jp

**Keywords:** hyperglycemia, obesity, *Boesenbergia rotunda*, pinostrobin, nitric oxide, insulin

## Abstract

Obesity, which is characterized by excessive body fat accumulation and energy imbalance, is a major risk factor for type 2 diabetes mellitus. *Boesenbergia rotunda* rhizomes (known as fingerroots) exhibit a variety of pharmacological activities and are used in traditional medicine. Pinostrobin was identified as a major constituent of *Boesenbergia rotunda* rhizome (BR) extract and suppressed nitric oxide production in interleukin 1β-treated hepatocytes. Next, we investigated the anti-obesity effects of BR extract in *ob/ob* mice, a type 2 diabetes mellitus model. The *ob/ob* mice were treated with or without 1% BR extract for 14 days and then analyzed for serum insulin and triglyceride levels, liver histology, and liver mRNA expression. The administration of BR extract significantly decreased blood glucose concentrations and increased serum insulin concentrations in *ob/ob* mice. In addition, this treatment reduced triglyceride levels in both the serum and liver and decreased lipid accumulation in hepatocytes. Microarray analysis revealed that hepatic mRNA affected the expression of genes involved in inflammation, lipid metabolism, lipid synthesis, and insulin signaling, leading to improvements in obesity. Because BR extract has hypoglycemic and antidiabetic effects on *ob/ob* mice, it might be a promising candidate for addressing obesity and related metabolic disorders.

## 1. Introduction

Obesity is characterized by the abnormal accumulation of ≥20% body fat, causing an individual to exceed their optimal body weight, and is caused by an imbalance in energy intake and expenditure [1]. Obesity is generally characterized by the accumulation of triglycerides, which play an important role as a source of energy, in the blood and tissues. Hypertriglyceridemia can lead to cardiovascular disorders [2]. Obesity has emerged as a leading global public health challenge, contributing to a high number of annual deaths. The Centers for Disease Control and Prevention (CDC) stated that more than one-third of adults are considered obese [3]. Globally, more than 2.1 billion individuals, from children to adults, are affected by obesity [4]. Childhood obesity has become a major public health problem, and obese children are likely to remain obese into adulthood and develop various diseases [5].

Obesity is closely associated with various diseases, such as type 2 diabetes mellitus (DM), hypertension, liver disorders, and cancer. Increasing fat intake under obesity conditions induces an increase in the amount of nonesterified fatty acids and proinflammatory cytokines, which can play a role in insulin resistance, leading to type 2 DM [6]. Under diabetic conditions, adipose tissue releases proinflammatory cytokines, leading to persistent low-grade inflammation. These cytokines also suppress insulin signaling by inhibiting insulin receptor tyrosine kinase activity [7]. Triglyceride accumulation in the liver can cause endoplasmic reticulum (ER) stress, which triggers the activation of the transcription factor NF-κB to promote the expression of the proinflammatory cytokines that contribute to insulin resistance. In addition, ER stress can induce the phosphorylation of insulin receptor substrate 1 (IRS-1), affecting the ability of cells to detect changes in blood insulin levels [8]. The accumulation of saturated fatty acids such as palmitate can induce inflammation and apoptosis in hepatocytes [9].

Fingerroot (*Boesenbergia rotunda* L.) is part of the ginger family (Zingiberaceae). This plant grows in Southeast Asia, India, and Sri Lanka. It is known as *Temu Kunci* in Indonesia and *Obangajutsu* in Japan. Its name comes from the characteristic globular-shaped, central subterraneous rhizomes, which resemble the fingers of a hand [10,11,12]. Traditionally, *B. rotunda* rhizomes have been used as a spice and traditional medicine [13]. *B. rotunda* rhizome (BR) extract has a variety of pharmacological properties, including anti-inflammatory, anticancer, and antioxidant properties [14,15]. BR extract reportedly has anti-obesity effects by inhibiting adipogenesis [16]. This anti-obesity activity may be attributed to pinostrobin, a major constituent of this plant. This constituent exhibited high adipogenic suppressor activity by inhibiting the phosphorylation of adipogenesis-related proteins by Akt, Jnk, and p38 kinase in the mouse adipocyte-like cell line 3T3-L1 [17]. In the human hepatoma cell line HepG2, pinostrobin reduces the transcription of convertase subtilisin/kexin type 9 (PCSK9) by modulating FoxO3a, resulting in an increase in low-density lipoprotein (LDL) uptake via the LDL receptor (LDLR) [18]. However, few reports have comprehensively explained the anti-obesity mechanism of BR extract.

Nitric oxide (NO) is a proinflammatory mediator that is induced in hepatocytes and macrophages in response to interleukin 1β (IL-1β) and lipopolysaccharide (LPS), respectively [19,20]. NO is synthesized by inducible nitric oxide synthase (iNOS, also known as NOS2). In rat hepatocytes, the expression of *iNOS* mRNA increased after the addition of IL-1β and the iNOS protein level increased after the addition of IL-1β, with NO production being correlated with the iNOS protein level [21,22].

In this study, the effects of BR extract and pinostrobin on inflammatory responses were analyzed in rat hepatocytes. The *ob/ob* mouse has mutations in the *leptin* gene, leading to hyperglycemia, hyperlipidemia, and insulin resistance [23]. These mice were used as an animal model of severe obesity and type 2 DM. Therefore, we investigated the effects of the hydrophobic fraction of BR extract by administering it to *ob/ob* mice. Transcriptomic microarray analysis was performed to clarify the mechanism of the effects of BR extract.

## 2. Results

### 2.1. BR Extract Suppressed NO Production in Rat Hepatocytes

**Figure 1:**
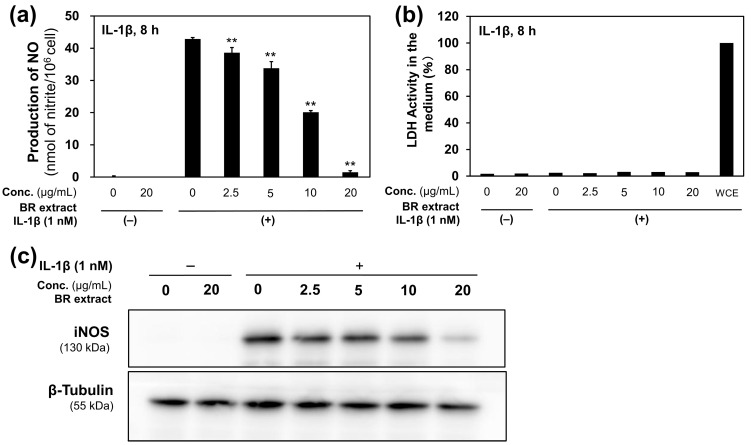
NO production in rat hepatocytes in the presence of BR extract and/or IL-1β (1 nM). (**a**) BR extract decreased NO levels. (**b**) BR extract had no cytotoxic activity, confirmed by the LDH activity of the medium. The LDH activity of the whole-cell extract (WCE) was set at 100% (positive control). (**c**) BR extract reduced iNOS expression in hepatocytes. NO levels in the medium are shown as the means ± standard deviations (SDs) (*n* = 3). ** *p* < 0.01 versus IL-1β alone.

To evaluate the anti-inflammatory activity of BR extract, NO production was monitored in primary cultured rat hepatocytes. The results showed that BR extract significantly reduced IL-1β-induced NO production in hepatocytes in a dose-dependent manner (Figure 1a). The extract also did not induce hepatocyte toxicity, as indicated by the lactate dehydrogenase (LDH) activity in the medium; this activity serves as a marker of damaged cells (Figure 1b). Western blotting analysis revealed that BR extract decreased iNOS expression in IL-1β-treated hepatocytes (Figure 1c). iNOS catalyzes the conversion of L-arginine to L-citrulline and NO [24]. BR extract reduced NO production and iNOS expression in IL-1β-treated rat hepatocytes, suggesting that BR extract may have anti-inflammatory effects.

### 2.2. Chemical Analysis of BR Extract and Identification of Pinostrobin

**Figure 2:**
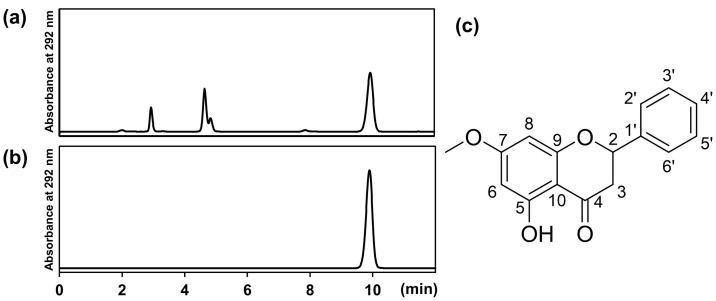
Chemical analysis of pinostrobin in BR extract. (**a**) HPLC chromatogram of BR extract. (**b**) HPLC chromatogram of (±)-pinostrobin (standard). (**c**) Chemical structure of pinostrobin.

It is expected that pinostrobin, a major constituent of BR extract, may be responsible for suppressing NO production in hepatocytes. To investigate this possibility, we first purified pinostrobin from its extract. High-performance liquid chromatography (HPLC) analysis of BR extract revealed a significant peak at a retention time of 9.85 min. This retention time closely corresponded to that of the standard compound pinostrobin, confirming its presence in the extract (Figure 2a,b). The peak areas of pinostrobin in the sample were used to calculate its content, accounting for 26.9% of the extract. This substantial concentration suggests that pinostrobin is a major constituent of BR extract. Methanol extract was purified by preparative thin-layer chromatography (TLC) to collect compound **1**, and further analysis was conducted via nuclear magnetic resonance (NMR) spectroscopy and optical rotation measurement.

Compound **1** exhibited the following features: pale yellow powder; ${\left[\alpha \right]}_{D}^{20}$ 0.6 (*c* 0.129, CHCl_3_); ^1^H NMR (500 MHz, CDCl_3_, ppm) δ 2.83 (1H, dd, *J* = 17.1, 3.0 Hz, H-3b), 3.09 (1H, dd, *J* = 17.1, 13.0 Hz, H-3a), 3.81 (3H, s, 7-OCH_3_), 5.43 (1H, dd, *J* = 13.0, 3.0 Hz, H-2), 6.07 (1H, d, *J* = 2.3 Hz, H-8), 6.08 (1H, d, *J* = 2.3 Hz, H-6), 7.42 (3H, m, H-3′, H-4′, and H-5′), 7.46 (2H, m, H-2′ and H-6′), 12.03 (1H, s, 5-OH); ^13^C NMR (125 MHz, CDCl_3_, ppm) δ 43.4 (C-3), 55.7 (7-OCH_3_), 79.2 (C-2), 94.3 (C-8), 95.1 (C-6), 103.1 (C-10), 126.2 (C-2′ and C-6′), 128.9 (C-3′, C-4′, and C-5′), 138.4 (C-1′), 162.8 (C-9), 164.1 (C-5), 168.0 (C-7), 195.8 (C-4). This compound was identified as (±)-pinostrobin (Figure 2c) on the basis of ^1^H and ^13^C NMR spectral analysis in comparison with previously published results [25].

### 2.3. (±)-Pinostrobin Suppresses NO Production in IL-1β-Treated Hepatocytes

**Figure 3:**
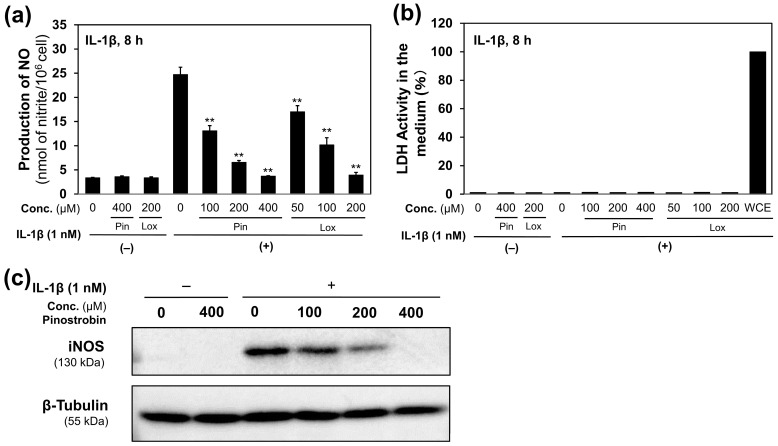
(±)-Pinostrobin decreased NO production in IL-1β-induced hepatocytes. (**a**) (±)-Pinostrobin (Pin) reduced NO levels compared with loxoprofen (Lox, a positive control). (**b**) (±)-Pinostrobin showed no cytotoxic activity, as confirmed by LDH levels in the medium. The whole-cell extract (WCE) of hepatocytes was assumed to have 100% LDH activity. (**c**) (±)-Pinostrobin decreased iNOS expression. NO levels in the medium are presented as the means ± SDs (*n* = 3). ** *p* < 0.01 versus IL-1β alone.

An NO production assay was performed to determine whether (±)-pinostrobin is a key compound for the suppression of NO production. As expected, (±)-pinostrobin significantly inhibited NO production in IL-1β-treated hepatocytes, with an IC_50_ value of 204.4 ± 76.8 µM (Figure 3a). The very low LDH activity in the medium indicated that (±)-pinostrobin was not cytotoxic to hepatocytes (Figure 3b). When the iNOS protein expression was examined by Western blot analysis, (±)-pinostrobin reduced IL-1β-induced iNOS protein expression in hepatocytes (Figure 3c).

### 2.4. Effects of BR Extract on Body Weight and Blood Glucose Levels in ob/ob Mice

**Figure 4:**
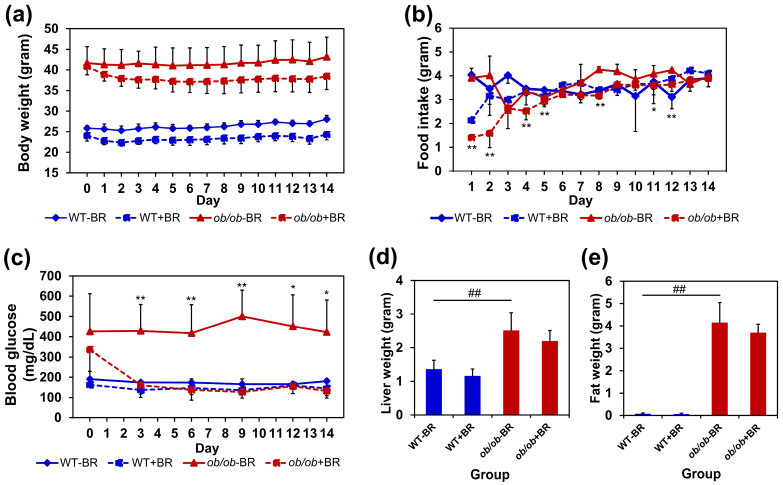
Effects of BR extract on *ob/ob* mice. (**a**) No significant differences in body weight were detected between the *ob/ob* + BR and *ob/ob*−BR mouse groups. (**b**) Food intake during the treatment period. (**c**) BR extract decreased blood glucose levels in *ob/ob* mice. The data represent the means ± SDs (*n* = 4 mice). * *p* < 0.5 and ** *p* < 0.01 compared with *ob/ob* − BR. There was no significant difference between *ob/ob*+/−BR mice in liver (**d**) and fat (**e**) weights. The data represent the means ± SDs (*n* = 4 mice). ^##^ *p* < 0.01 versus the WT−BR group.

Next, BR extract was administered to *ob/ob* mice to examine its anti-obesity activity. The body weight of *ob/ob* mice treated with BR extract (*ob/ob* + BR group) was lower than that of *ob/ob* mice not treated with BR extract (*ob/ob* − BR group). Although the difference was not statistically significant, it may have been due to variations in food intake at the beginning of the treatment period (Figure 4a,b).

The blood glucose concentrations of *ob/ob* + BR mice were significantly lower than those of *ob/ob* − BR mice (Figure 4c). On day 14, blood glucose levels were recorded at 132 mg/dL in *ob/ob* + BR mice and 422 mg/dL in *ob/ob* − BR mice. In contrast, the blood glucose levels of wild-type (WT) mice treated with or without BR extract did not significantly change. These results suggest that BR extract has hypoglycemic activity. The liver and fat weights did not significantly differ between the groups treated with and without BR extract (Figure 4d,e).

### 2.5. BR Extract Increases Serum Insulin Levels

**Figure 5:**
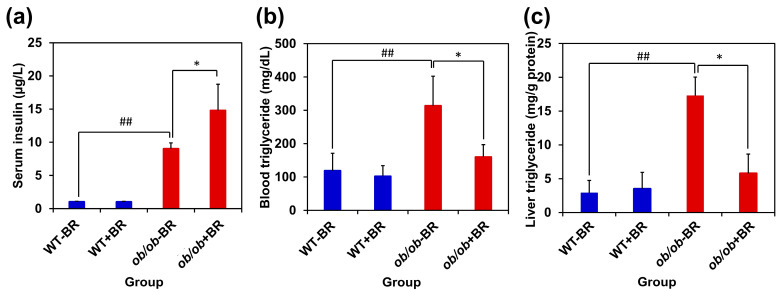
Insulin and triglyceride concentrations in *ob/ob* mice treated with BR extract. (**a**) Elevated serum insulin levels in *ob/ob* + BR mice compared with *ob/ob* − BR mice. (**b**) Decreased serum triglyceride concentration caused by BR extract. (**c**) Decreased triglyceride concentration in the liver. The protein content was normalized to the protein content of the liver. The data represent the means ± SDs (*n* = 4 mice). * *p* < 0.05 versus the *ob/ob* − BR group. ^##^ *p* < 0.01 versus the WT−BR group.

We measured blood insulin levels to evaluate the antidiabetic activity of BR extract. There were no significant differences in blood sugar levels between WT mice treated with or without BR extract. However, *ob/ob* + BR mice exhibited a significant increase in blood insulin levels compared with *ob/ob* − BR mice (Figure 5a). These results indicate that BR extract increases insulin production in *ob/ob* mice.

### 2.6. BR Extract Reduces Serum and Liver Triglyceride Levels

Next, we measured the serum and liver triglyceride levels. Triglycerides are lipids that serve as the main energy storage, transported in the blood and stored in adipose tissues [26]. The serum triglyceride concentrations of *ob/ob* + BR mice were significantly lower than those of *ob/ob* − BR mice (Figure 5b). The triglyceride content in the liver was also significantly lower in *ob/ob* + BR mice than in *ob/ob* − BR mice (Figure 5c). In contrast, there was no significant difference between WT mice with or without the administration of BR extract (WT + BR group versus WT − BR group). These data suggest that BR extract effectively reduces high triglyceride levels in the serum and liver of *ob/ob* mice but does not affect triglyceride levels in wild-type mice.

### 2.7. The Administration of BR Extract Reduced Lipid Accumulation in the Liver

**Figure 6:**
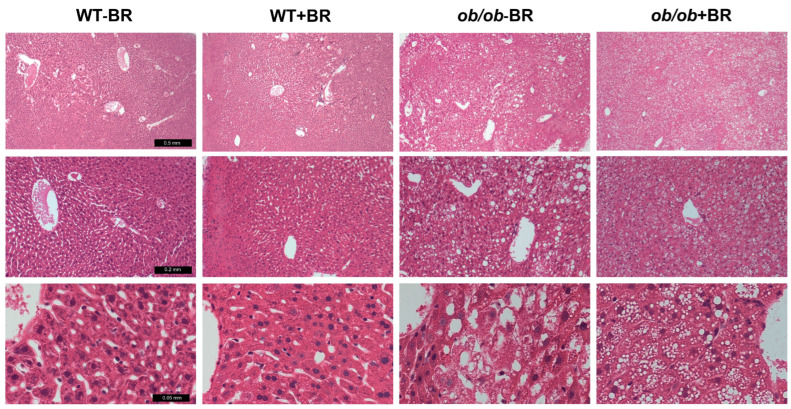
Hematoxylin and eosin staining of the livers of wild-type (WT) and *ob/ob* mice fed a standard diet without/with BR extract. Lipid droplets in hepatocytes dissolved during deparaffinization and became white after staining. From the upper line: low- (40×), middle- (100×), and high- (400×) magnification images with scale bars of 0.5, 0.2, and 0.05 mm, respectively.

To examine lipid accumulation in the liver, hematoxylin and eosin staining was performed on liver tissue (Figure 6). The livers of wild-type (WT) mice treated with and without BR extract presented an intact lobular architecture around the central vein. In contrast, *ob/ob* − BR mice presented numerous lipid droplets within the cytoplasm of hepatocytes, while *ob/ob* + BR mice presented fewer droplets. These lipid droplets constituted an inert storage of triglycerides in liver tissue [27].

### 2.8. Hepatic mRNA Expression Profiles of ob/ob Mice

Microarray mRNA expression analysis revealed distinct mRNA expression profiles in the livers of *ob/ob* + BR and *ob/ob* − BR mice. Among the 28,847 genes analyzed, 1087 genes (3.77%) presented significant changes in mRNA expression in the *ob/ob* + BR group compared with the *ob/ob* − BR group: the mRNA expression of 512 genes (1.77%) was upregulated, and that of 575 genes (2.00%) was downregulated in the *ob/ob* + BR group. The differentially expressed genes were further classified according to their roles in inflammation, lipid metabolism, and insulin signaling based on the Kyoto Encyclopedia of Genes and Genomes (KEGG) pathway and Gene Ontology (GO) annotation.

### 2.9. BR Extract Suppresses the Expression of mRNAs Associated with Inflammatory Responses

BR extract significantly affected inflammatory pathways in the livers of *ob/ob* mice. As shown in Table 1, the genes involved in NO biosynthesis were significantly downregulated, which may have caused a reduction in NO production. Accordingly, several genes involved in NF-κB activation, including the *Ticam1* gene, were downregulated. The expression of the *Tnfaip3* gene, whose protein acts as a negative regulator of the NF-κB signaling pathway, was upregulated. The reduced expression of the *Stat1* and *Stat2* genes in the JAK-STAT pathway was also downregulated, which may have attenuated inflammatory responses in *ob/ob* mice.

### 2.10. Effects of BR Extract on the Expression of mRNAs Related to Lipid Metabolism and Insulin Signaling

Next, we examined the genes involved in fatty acid metabolism and triglyceride homeostasis. As shown in Table 2, the mRNA expression of the *Acnat1* and *Acnat2* genes was decreased in the livers of *ob*/*ob* − BR mice. These enzymes are crucial for the fatty acid metabolism for fatty acid oxidation and processing. Concurrently, the expression of genes encoding enzymes involved in lipid synthesis and storage (*Aacs*, *Alkbh7*, and *Cyp4a12b*) was downregulated.

We further investigated the expression of genes involved in insulin signaling in the livers of *ob*/*ob* − BR mice (Table 3). The administration of BR extract significantly altered the mRNA expression of several key genes, such as the *Igfbp2* and *Cga* genes, which may modulate insulin signaling pathways.

## 3. Discussion

BR is a traditional herbal medicine known for its potent anti-obesity effects, to which its constituent pinostrobin may be primarily attributed. We first identified pinostrobin purified from BR extract as (±)-pinostrobin, i.e., a racemic mixture of *R* and *S* enantiomers. The pharmacological activity of pinostrobin may be attributed to its ketone group at the four-position and a hydroxy group at the five-position [28] (Figure 2c). The content of pinostrobin was estimated to be 26.9% of BR extract, confirming that pinostrobin is a major constituent of this crude drug. Furthermore, (±)-pinostrobin clearly inhibited NO production, with an IC_50_ of 204 µM (=55.3 µg/mL) in IL-1β-induced hepatocytes (Figure 3a). Pinostrobin inhibits cyclooxygenase-2 (COX-2) and 5-lipoxygenase (5-LOX) [29], both of which produce proinflammatory mediators.

Given the content of pinostrobin in BR extract and the IC_50_ for the suppression of NO production, it is difficult to explain the activity of BR extract solely by pinostrobin. Pinocembrin, another constituent of BR extract, has also been reported to reduce NO production and iNOS expression in a macrophage line and a microglial cell line [28,30]. Therefore, both pinostrobin and pinocembrin may contribute to the anti-inflammatory effects of BR extract.

mRNA expression analysis revealed that BR extract modulated the expression of genes involved in NO biosynthesis, NF-κB signaling, and the JAK-STAT pathway in the livers of *ob/ob* mice (Table 1). The downregulation of *Ass1* gene expression by BR extract results in a reduction in NO levels [31]. Because Tnfaip3 encodes a ubiquitin-editing enzyme that functions as an NF-κB inhibitor [32], upregulated *Tnfaip3* gene expression may suppress NF-κB signaling. In addition, downregulated expression of the *Ticam1* gene, whose protein facilitates the translocation of NF-κB to the nucleus [33], may lead to the inhibition of NF-κB signaling. Downregulation of *Stat1* and *Stat2* gene expression by BR extract may decrease inflammation [34]. Taken together, these findings suggest that BR extract may inhibit inflammatory responses through pathways mediated by the transcription factors NF-κB and Stat. However, while gene expression data strongly suggest anti-inflammatory effects, additional assays could further clarify the underlying mechanisms. Future studies should consider these assays to validate these findings.

Because inflammation is one of the causes of obesity [8], BR extract effectively improved the symptoms of *ob/ob* mice. Indeed, BR extract decreased serum triglyceride levels and hepatic triglyceride accumulation in *ob/ob* mice, possibly due to changes in the expression of key genes involved in lipid metabolism, especially the expression of the *Acnat1* and *Acnat2* genes, which was significantly increased in the livers of *ob/ob* mice treated with BR extract (*ob/ob* + BR) (Table 2). Acnat1 and Acnat2 are crucial enzymes for degrading fatty acids to produce energy [35]. Additionally, the mRNA levels of the acetoacetyl-CoA synthetase (*Aacs*) gene, which encodes an enzyme critical for the conversion of acetoacetate into acetoacetyl-CoA in fatty acid synthesis, are downregulated [36]. Overall, BR extract may lead to decreased serum and liver triglyceride levels in *ob/ob* mice.

The insulin signaling pathway may be regulated by BR extract (Table 3). Our mRNA expression analysis suggested that BR extract increased the expression of key proteins such as insulin-like growth factor-binding protein 2 (IGFBP2) and β-2 adrenergic receptor (Adrb2). IGFBP2 binds to insulin-like growth factor (IGF) and promotes the uptake and utilization of glucose in tissues [37], resulting in the inhibition of adipogenesis and lipogenesis in human visceral tissue [38]. Because Adrb2 also activates protein kinase A (PKA), upregulation of Adrb2 may augment the effects of insulin [39]. These changes in mRNA expression suggest that BR extract may exert anti-obesity effects by affecting the expression of insulin signaling-related genes.

As mentioned above, the present study revealed the anti-inflammatory, anti-obesity, and insulin-sensitizing effects of BR extract. It is necessary to clarify which compounds in BR extract are responsible for these effects. It is likely that pinostrobin, a major and principal constituent of BR extract, plays a pivotal role in this effect, although its anti-inflammatory activity does not directly correlate with other pharmacological activities. Pinocembrin, a constituent of BR extract, may be another candidate. More studies are needed to answer this question. Future studies should provide deeper insights into the mechanisms underlying the pharmacological effects of BR extract and its constituents, as well as the utilization of BR extract for the treatment of obesity and obesity-associated metabolic disorders.

## 4. Materials and Methods

### 4.1. General Experimental Procedures

A JNM-ECS500 NMR spectrometer (JEOL Ltd., Akishima, Tokyo, Japan) operating at 500 MHz (^1^H) and 125 MHz (^13^C) was used to record the NMR spectra. Deuterated chloroform (CDCl_3_) was purchased from Eurisotop (Saint-Aubin, France). The optical rotations of the compounds were measured via a DIP-370 polarimeter (JASCO Corporation, Hachioji, Tokyo, Japan).

### 4.2. Plant Materials and Extraction

BRs were obtained from the UPT Herbal Materia Medika Batu Laboratory in Malang, East Java, Indonesia, via a specimen voucher (190612.TKC.LR.001). One kilogram of rhizome powder was macerated twice with 96% methanol (1:10, *w/v*) at 50 °C for 1 h. The extract was filtered through Whatman filter paper and subsequently evaporated at 50 rpm and 37 °C using a Buchi R-210 Rotavapor System (Buchi Corporation, New Castle, DE, USA).

### 4.3. Quantification of Pinostrobin Content

In accordance with a previously established method [40], HPLC analysis was performed to measure the content of pinostrobin, which was eluted at a flow rate of 1.0 mL/min using a mixture of acetonitrile, methanol, and 0.05% phosphate acid in water (40:25:35) and detected at a wavelength of 292 nm. A single peak with a retention time of 9.85 min corresponded to pinostrobin. As a standard compound, pinostrobin (purity, ≥99.0%; Sigma–Aldrich Corporation, St. Louis, MO, USA) was accurately weighed and dissolved in methanol to make a stock solution of 1.17 mg/mL; 2.5, 5.0, and 10.0 μL from each injection were analyzed in triplicate. The calibration curve of the standard compound was calculated by plotting the peak areas (*y*) against a series of injection amounts (*x*, μg), and the regression equation was *y* = 2,924,211 *x* (*R*^2^ = 0.9986). The methanol extract was accurately weighed and dissolved in methanol to prepare a sample solution of 2.53 mg/mL, and the sample solution (10.0 µL) was analyzed in triplicate. The peak areas of pinostrobin in the sample solution were fitted to the calibration curve, and the amount of pinostrobin in 10.0 µL of the sample solution was calculated. Because the amount of pinostrobin in 10.0 µL of the sample mixture (25.3 µg of methanol extract) was calculated to be 6.8 µg, the content of pinostrobin in the methanol extract was 26.9%.

### 4.4. Primary Cultured Rat Hepatocytes

Male Wistar rats from Charles River Laboratories Japan, Inc. (Yokohama, Japan) were maintained at 21–23 °C with a 12 h alternating light–dark cycle. They were fed a γ-ray-irradiated CRF-1 diet and had unlimited access to water. After a 1-week acclimation period, hepatocytes were extracted from their livers via collagenase perfusion according to a previously published method [41]. Hepatocytes were resuspended in Williams’ E medium (Sigma–Aldrich Corp.), seeded at 1.2 × 10⁶ cells per 35 mm culture dish, and incubated at 37 °C overnight. Next, the cells were treated with 1 nM recombinant rat IL-1β and either an extract or a compound for 8 h. The animal care procedures and experiments were conducted according to the guidelines of the Japanese Government and were approved by the Animal Care Committee of Ritsumeikan University, Biwako-Kusatsu Campus (No. BKC2021-031).

### 4.5. NO Level Assessment and LDH Activity Analysis

Hepatocytes were exposed to BR extract, pinostrobin, or loxoprofen sodium for 8 h at 37 °C, with or without the addition of 1 nM recombinant rat IL-1β [41]. NO levels in the medium were quantified via the Griess technique [42]. After a 5 min incubation at room temperature, the absorbance was recorded at 540 nm. Cytotoxicity was evaluated by assessing LDH activity in the medium via a Cytotoxicity LDH Assay Kit-WST (Dojindo Molecular Technologies Inc., Kumamoto, Japan), whereas whole-cell extracts were obtained by lysing cells with 2% (*w/v*) Triton X-100. The IC_50_ values were calculated unless cytotoxic effects were observed [43].

### 4.6. Western Blot Analysis

Hepatocytes were treated with 1 nM IL-1β, BR extract, pinostrobin, or loxoprofen sodium for 8 h at 37 °C, and whole-cell lysates were then prepared. Hepatocytes were lysed, and proteins were separated via sodium dodecyl sulfate–polyacrylamide gel electrophoresis before being transferred onto a Sequi-Blot membrane (Bio-Rad, Hercules, CA, USA). Immunostaining was conducted using mouse monoclonal antibody against rat iNOS (1:1000) (Thermo Fisher Scientific, Waltham, MA, USA) and rabbit polyclonal antibody against rat β-tubulin (1:1000) (Cell Signaling Technology Inc., Danvers, MA, USA). The secondary antibodies used were horse radish peroxidase-conjugated anti-mouse IgG (1:5000) for iNOS (Cell Signaling Technology Inc., Danvers, MA, USA) and horse radish peroxidase-conjugated anti-rabbit IgG (1:1000) for β-tubulin (Cell Signaling Technology Inc., Danvers, MA, USA). The proteins were then visualized using an Enhanced Chemiluminescence Blotting Detection Reagent (Cytiva, Tokyo, Japan).

### 4.7. Administration of BR Extract to Mice

Male *Lep^ob/ob^* (*ob/ob*) and *Lep*^+/+^ (wild-type, WT) mice (7 weeks old) were obtained from Japan SLC, Inc. (Hamamatsu, Japan) and maintained in a controlled environment at 21–23 °C under a 12 h light–dark cycle, with free access to a γ-ray-irradiated CRF-1 diet and water. After 1 week of acclimation, each mouse type, consisting of eight individuals, was randomly assigned to two groups of four and received either a γ-ray-irradiated CRF-1 diet with or without 1% BR extract for 14 days [44]. The groups were designated as WT-BR (25.85 ± 0.64 g), WT+BR (24.03 ± 1.32 g), *ob/ob* − BR (41.67 ± 4.00 g), and *ob/ob* + BR (40.84 ± 2.04 g). Blood glucose levels were measured every 3 days from a small tail incision using a GLUCOCARD PlusCare GT-1820 Monitoring System (Arkray, Inc., Kyoto, Japan). On day 14, the mice were euthanized via isoflurane inhalation, and liver and blood samples were collected for various analyses.

### 4.8. Measurement of Insulin and Triglyceride Levels

Blood was collected from the heart, and the insulin and triglyceride levels in the serum were quantified via an enzyme-linked immunosorbent assay (Mercodia AB, Uppsala, Sweden) and LabAssay Triglyceride Kits (FUJIFILM Wako Pure Chemical Corporation, Osaka, Japan), respectively. Liver samples (ca. 100 mg) were homogenized using a Polytron homogenizer (Kinematica AG, Luzern, Switzerland) in 20 volumes of a chloroform–methanol mixture (2:1 *v*/*v*) following a previously described method [45,46]. The total extracted lipids were washed and measured via LabAssay kits.

### 4.9. Liver Tissue Histology

The livers from euthanized mice were fixed in 4% formaldehyde. Liver specimens were embedded in paraffin. Thin sections from the blocks were deparaffinized, stained with hematoxylin and eosin, and examined according to a previously established protocol [47].

### 4.10. Total RNA from the Liver

The liver tissue of *ob/ob* mice was lysed in Sepasol I Super G Solution (Nacalai Tesque Inc., Kyoto, Japan) using a Polytron homogenizer [37]. Total RNA was isolated using previously established methods [44,48]. Briefly, chloroform was added to the lysate and centrifuged, and total RNA was precipitated from the aqueous layer with isopropanol and purified using an RNAqueous Kit and a TURBO DNA-free kit (Thermo Fisher Scientific, Waltham, MA, USA).

### 4.11. Microarray Analysis of mRNA Expression

The total RNA extracted from mouse livers was combined for each group (3 mice per group), and microarray mRNA expression analysis was performed according to a previously published method [49]. Briefly, cyanine 3 (Cy3)-labeled complementary RNA was hybridized to the SurePrint G3 Mouse Gene Expression 8 × 60K Microarray ver. 2 (Agilent Technologies, Santa Clara, CA, USA) and scanned to obtain signal intensities. Each probe set produced two signals: one from the Cy3-labeled cRNA of *ob/ob* + BR mice (‘Experiment’) and one from *ob/ob* – BR mice (‘Base’). Probe sets with both signals detected (score = 2) were analyzed. Probe sets with very low signals (<100) were excluded. Only reliable signals were used for analysis and classified via the KEGG pathway (https://www.genome.jp/kegg/pathway.html) (accessed on 10 August 2024) and GO annotations (https://geneontology.org) (accessed on 10 August 2024). Signal ratios were calculated by comparing *ob/ob* + BR and *ob/ob* – BR mice with fold changes. Probe sets with a ratio ≤ 0.5 and a ratio ≥ 2.0 were considered significant.

### 4.12. Statistical Analysis

The figures show representative results from independent experiments with similar findings. The values are presented as the means ± SDs. Differences were analyzed via Student’s *t* test followed by Bonferroni correction using Microsoft Excel 2016. The significance was set to 0.05 and 0.01.

## 5. Conclusions

An extract from BRs and its major constituent pinostrobin markedly reduced NO production in IL-1β-treated hepatocytes. We identified pinostrobin in BR extract as (±)-pinostrobin, which appears to be responsible for the anti-inflammatory effects. When this extract was administered to *ob*/*ob* mice, a type 2 DM model, a marked hypoglycemic effect, and an increase in serum insulin levels were observed. BR extract effectively reduced the serum triglyceride concentration and hepatic triglyceride accumulation. In the livers of *ob*/*ob* mice, BR extract decreased the expression of genes involved in inflammatory responses and NO biosynthesis and increased the expression of genes promoting lipid metabolism and insulin signaling. This study provides a basis for the treatment of obesity and obesity-associated metabolic disorders using BR extract.

## Figures and Tables

**Table 1:** Hepatic expression of mRNAs associated with inflammatory responses.

Gene Description	Gene Symbol	Signal Ratio of BR(+)/BR(−)
**Nitric oxide (NO) biosynthesis**		
***Decreased:***		
TIR domain-containing adaptor molecule 1	*Ticam1*	0.446
Argininosuccinate synthetase 1	*Ass1*	0.420
Histocompatibility 2, M region locus 3	*H2-M3*	0.353
**NF-** **κB activity and signaling**		
***Increased:***		
Tumor necrosis factor, alpha-induced protein 3	*Tnfaip3*	3.184
***Decreased:***		
Signal transducer and activator of transcription 1	*Stat1*	0.450
TIR domain-containing adaptor molecule 1	*Ticam1*	0.446
Tripartite motif-containing 37	*Trim37*	0.432
Leukocyte immunoglobulin-like receptor, subfamily B, member 4A	*Lilrb4a*	0.426
**JAK-STAT cascade**		
***Decreased:***		
Signal transducer and activator of transcription 1	*Stat1*	0.450
Signal transducer and activator of transcription 2	*Stat2*	0.376

**Table 2:** Hepatic expression of mRNAs related to lipid metabolism.

Gene Description	Gene Symbol	Signal Ratio of BR(+)/BR(−)
**Fatty acid metabolic process**		
***Increased:***		
Acyl-coenzyme A (CoA) amino acid *N*-acyltransferase 2	*Acnat2*	2.991
Acyl-coenzyme A (CoA) amino acid *N*-acyltransferase 1	*Acnat1*	2.731
***Decreased:***		
Acetoacetyl-CoA synthetase	*Aacs*	0.384
AlkB homolog 7	*Alkbh7*	0.315
Cytochrome P450, family 4, subfamily a, polypeptide 12B	*Cyp4a12b*	0.236
**Triglyceride homeostasis**		
***Increased:***		
Apolipoprotein A-I	*Apoa1*	3.211
Adenosine A1 receptor	*Adora1*	2.912

**Table 3:** Hepatic expression of mRNAs related to the insulin signaling pathway.

Gene Description	Gene Symbol	Signal Ratio of BR(+) versus (−)
**Insulin signaling pathway**		
***Increased:***		
Insulin-like growth factor binding protein 2	*Igfbp2*	3.469
Adenosine A1 receptor	*adora1*	2.912
Adrenergic receptor, beta 2	*Adrb2*	2.555
***Decreased:***		
Glycoprotein hormones, alpha subunit	*Cga*	0.421

## Data Availability

The data presented in this study are available upon request from the corresponding author.
